# Enhanced CXCR4 Expression Associates with Increased Gene Body 5-Hydroxymethylcytosine Modification but not Decreased Promoter Methylation in Colorectal Cancer

**DOI:** 10.3390/cancers12030539

**Published:** 2020-02-26

**Authors:** Alexei J. Stuckel, Wei Zhang, Xu Zhang, Shuai Zeng, Urszula Dougherty, Reba Mustafi, Qiong Zhang, Elsa Perreand, Tripti Khare, Trupti Joshi, Diana C. West-Szymanski, Marc Bissonnette, Sharad Khare

**Affiliations:** 1 Department of Medicine, Division of Gastroenterology and Hepatology, University of Missouri, Columbia, MO 65212, USAbeefjiao@gmail.com (Q.Z.); elpxc9@mail.missouri.edu (E.P.); kharet@health.missouri.edu (T.K.); 2 Department of Preventive Medicine and The Robert H. Lurie Comprehensive Cancer Center, Northwestern University Feinberg School of Medicine, Chicago, IL 60611, USA; wei.zhang1@northwestern.edu; 3 Department of Medicine, University of Illinois, Chicago, IL 60607, USA; zhangxu@uic.edu; 4 Bond Life Sciences Center, University of Missouri, Columbia, MO 65201, USA; zengs@mail.missouri.edu (S.Z.); joshitr@health.missouri.edu (T.J.); 5 Department of Electrical Engineering and Computer Science, University of Missouri, Columbia, MO 65201, USA; 6 Department of Medicine, Section of Gastroenterology, Hepatology and Nutrition, The University of Chicago, Chicago, IL 60637, USA; udougher@medicine.bsd.uchicago.edu (U.D.); rmustafi@uchicago.edu (R.M.); dcwest@bsd.uchicago.edu (D.C.W.-S.); mbissonn@medicine.bsd.uchicago.edu (M.B.); 7 Institute for Data Science and Informatics, University of Missouri, Columbia, MO 65211, USA; 8 Department of Health Management and Informatics, School of Medicine, University of Missouri, Columbia, MO 65212, USA; 9 Harry S. Truman Memorial Veterans’ Hospital, Columbia, MO 65201, USA

**Keywords:** colorectal cancer, DNA methylation, epigenetic regulation, CXCR4 gene expression, 5-hydroxymethylcytosine

## Abstract

In colorectal cancer (CRC), upregulation of the C-X-C motif chemokine receptor 4 (CXCR4) is correlated with metastasis and poor prognosis, highlighting the need to further elucidate *CXCR4*’s regulation in CRC. For the first time, DNA methylation and 5-hydroxymethylcytosine aberrations were investigated to better understand the epigenetic regulation of *CXCR4* in CRC. CXCR4 expression levels were measured using qPCR and immunoblotting in normal colon tissues, primary colon cancer tissues and CRC cell lines. Publicly available RNA-seq and methylation data from The Cancer Genome Atlas (TCGA) were extracted from tumors from CRC patients. The DNA methylation status spanning *CXCR4* gene was evaluated using combined bisulfite restriction analysis (COBRA). The methylation status in the *CXCR4* gene body was analyzed using previously performed nano-hmC-seal data from colon cancers and adjacent normal colonic mucosa. CXCR4 expression levels were significantly increased in tumor stromal cells and in tumor colonocytes, compared to matched cell types from adjacent normal-appearing mucosa. *CXCR4* promoter methylation was detected in a minority of colorectal tumors in the TCGA. The CpG island of the *CXCR4* promoter showed increased methylation in three of four CRC cell lines. CXCR4 protein expression differences were also notable between microsatellite stable (MSS) and microsatellite instable (MSI) tumor cell lines. While differential methylation was not detected in *CXCR4*, enrichment of 5-hydroxymethylcytosine (5hmC) in *CXCR4* gene bodies in CRC was observed compared to adjacent mucosa.

## 1. Introduction

C-X-C motif chemokine ligand 12 (CXCL12), and its cognate receptor C-X-C motif chemokine receptor 4 (CXCR4), constitutes a major cytokine-signaling pathway involved in both health and disease [1,2]. *CXCR4* deletion is embryonic lethal, emphasizing its essential role in normal development [3]. In addition, CXCR4 overexpression promotes tumorigenesis and its expression and activity are associated with cell proliferation [4,5,6,7,8], invasion [8,9,10,11,12,13], migration [8,14,15,16,17,18,19,20], inflammation [21], angiogenesis [22], and metastasis [23,24,25,26,27,28,29,30] in several cancer models. These myriad roles reflect the fact that CXCR4/CXCL12 signaling pathways can be activated by both autocrine and paracrine signaling pathways (e.g., via stromal (CXCL12)-epithelial (CXCR4) crosstalk). In the case of tumor metastasis, CXCL12 is released from stromal cells within the metastatic microenvironment, which in turn activates CXCR4 on cancer cells to increase cancer cell adhesion, motility and invasion in colon cancer cells [8]. Indeed, CXCR4 expression is an independent prognostic marker for several malignancies including: AML, multiple myeloma, squamous cell carcinoma, gastric cancer, renal cancer, hepatocellular carcinoma, and colorectal cancer (CRC) [31,32,33,34,35,36,37]. In the case of CRC patients, a meta-analysis correlated high CXCR4 expression with liver and lymph node metastasis and ultimately a reduction in overall survival [38].

Although the functions of CXCR4 are well described in tumor growth and invasion, the regulation of *CXCR4* gene expression in cancer is less well understood. While upstream loss-of-function mutations of the von Hippel-Lindau tumor suppressor gene (*VHL*) or aberrant signaling may result in CXCR4 overexpression in some cancers [39,40], other studies point to epigenetic regulation of *CXCR4*. For example, cytosine methylation (5mC) occurs widely in cancers including CRC [41,42,43,44,45,46]. Prior studies have linked increased *CXCR4* promoter cytosine methylation (5mC) to decreased CXCR4 protein expression in several non-colonic cancers such as primary breast cancer [29], decreased mRNA and protein in melanoma cell lines [47] and primary cervical cancer [48]. Increased CXCR4 expression was achieved with the knock-down of DNA methyltransferase 1 (DNMT1) or DNA methyltransferase 3 beta (DNMT3B), or with the inhibition of DNA methylation by 5-aza-2-deoxycytidine (5-aza) in pancreatic cancer cells [49]. Similarly, 5-aza treatment increased CXCR4 mRNA and protein levels in melanoma cells, and concomitantly enhanced cell migration [47]. These studies suggest that 5mC is an epigenetic silencer of *CXCR4* in cancer. Another epigenetic marker is 5-hydroxymethylcytosine (5hmC), which is either stable or serves as an “intermediate” of an active demethylation process [50]. Gene expression is commonly associated with 5hmC deposition in gene bodies [51] and differential 5hmC mapping in several cancers, including CRC has been achieved [52]. With these reports in mind, we sought to explore 5mC and 5hmC and CXCR4 upregulation in CRC. We hypothesized that enhanced *CXCR4* gene expression in CRC could be the result of epigenetic modifications, either through demethylation of the *CXCR4* promoter or an increase in 5hmC modification in the *CXCR4* gene body.

While past studies utilized immunohistochemistry in determining CXCR4 protein expression in human CRC, we combined proteomic and transcriptomic approaches of Western blotting and RT-PCR with 5mC combined bisulfite restriction analysis (COBRA) [53] to measure CXCR4 protein and mRNA expression along with promoter methylation status in CRC tumors. Moreover, for the first time, normal stromal cells and tumor-associated stromal cells were investigated for CXCR4 mRNA and protein expression. In addition, *CXCR4* analyses using publicly available databases of CRC transcriptomes, methylomes and 5-hydroxymethylomes were conducted to address our hypothesis. Herein, for the first time, we report that while 5mC distribution in the *CXCR4* promoter was not significantly changed in DNA from primary colon cancer tissue (compared to control tissue DNA), we observed that enhanced CXCR4 expression in CRC associates with increased 5hmC deposition in the gene body.

## 2. Results

### 2.1. CXCR4 Is Highly-Expressed in Colorectal Cancers

A prior study reported CXCR4 protein was upregulated in CRC compared to colonic mucosa and colonic adenomas [8]. In other studies, CXCR4 mRNA and protein were shown to be upregulated in metastatic tissue of the liver and lymph nodes compared to primary CRC tumors [30,37]. However, in the prior studies, it was not determined if CXCR4 overexpression correlated with CXCR4 in tumor colonocytes and/or in tumor stromal cells. Therefore, to extend our understanding of CXCR4 regulation in colorectal cancer, we separated colonocytes from stromal cells and examined their CXCR4 expression levels compared to their control counterparts.

**Figure 1:**
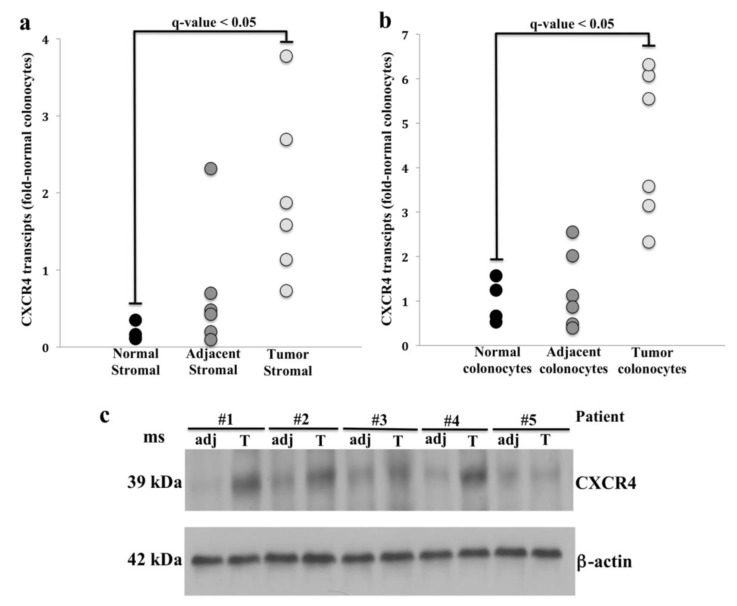
C-X-C motif chemokine receptor 4 (CXCR4) transcript and protein expression in tumor and adjacent normal tissue. RT-PCR analysis of *CXCR4* transcript abundance in normal and tumor tissue: (**a**) stromal cells and (**b**) colonocytes after multiple testing correction (Tukey post hoc test, Supplementary Table S1). Note the increase of *CXCR4* transcripts in both tumor stromal cells and colonocytes compared to adjacent matched tissue controls (*q*-value < 0.05). (**c**) CXCR4 protein expression in tumor colonocytes (T) and normal colonocytes adjacent to tumor (adj). Note the increased CXCR4 protein expression in tumor colonocytes compared to adjacent colonocytes from normal appearing mucosa (*n* = 5; *p*-value < 0.05, paired Student’s *t*-test, Supplementary Table S2).

We first examined *CXCR4* mRNA levels in colon tissues from four healthy controls and six CRC patients. We found in each case that colonocytes from normal colon, adjacent colon and cancers more strongly expressed *CXCR4* compared to stromal cells matched to the same tissue (*q*-value < 0.05) (Figure 1a,b). While *CXCR4* expression was lower in stromal cells, tumor-associated stromal cells expressed *CXCR4* more strongly than normal stromal cells (Figure 1a).

We next evaluated CXCR4 protein expression in tumor colonocytes from primary freshly isolated colorectal tumors and colonocytes from matched adjacent normal-appearing colonic mucosa. Overall, CXCR4 protein expression correlated with qPCR findings with increased CXCR4 protein in tumor colonocytes compared to colonocytes from adjacent mucosa (Figure 1c)**.** To assess the effects of cancer stage on *CXCR4* expression, we analyzed RNA-seq data in *n* = 1625 CRC patients from the TCGA. Normalized *CXCR4* transcript reads (FPKM values) served as the basis for comparing mRNA expression differences among the CRC that were categorized into 4 tumor stages. We found that there was a wide variation in the FPKM distribution within each tumor stage, ranging from low expression (FPKM value = 1) to high expression (FPKM value = 61). However, when comparing mean FPKM values, we did not find statistically significant differences among tumor stages (Supplementary Figure S1).

### 2.2. CXCR4 Promoter Hypomethylation Is Associated with Metastatic CRC

**Figure 2:**
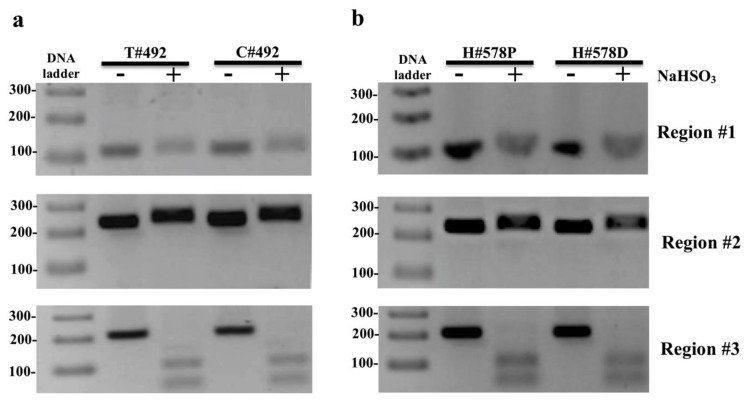
Representational combined bisulfite restriction analysis (COBRA) results of 3 regions of interest located in *CXCR4* from one colorectal cancer (CRC) patient: (**a**) (#492) and one healthy individual (Supplementary Figure S3) (**b**) (#578) (Supplementary Figure S4). Each sample was run on two lanes: lane 1) bisulfite-treated PCR amplified DNA without restriction enzyme digestion, which served as a reference control for unmethylated CpG and lane 2) bisulfite-treated PCR amplified DNA digested with a restriction enzyme recognizing amplicons containing a 5’mCpG sequence. Region #3 shows complete PCR product digestion, indicating that region #3 possesses cytosine methylation (5mC) *CXCR4* in tumor colonocytes (T# patient ID number), adjacent normal colonocytes (C# patient ID number), and proximal/distal colon of healthy individuals (H# patient ID number P/D proximal/distal colon). Restriction enzyme digestion was not evident in regions #1 or #2 in any tumor or normal colonocytes from any CRC patient or controls and therefore unmethylated in both regions. PCR product treated with restriction enzyme lane = +; PCR product from untreated sample lane = −; Restriction enzyme sensitive digestion was not detected for the amplicons of the intragenic CpG island region #2 or promoter region #1 in either tumor or control samples, indicating the absence of 5mC in those regions (Figure 2a). In addition to CRC samples, regions #1–#3 were assessed in colonocytes from proximal and distal colon in five healthy control individuals. The 5mC results of *CXCR4* for colonocytes from healthy control individuals were identical to those of malignant colonocytes from CRC patients, with 5mC present in region #3, but absent in regions #1 or #2 (Figure 2b). Whole COBRA gel images of region #1–#3 for the other CRC and normal control colons tested for *CXCR4* 5mC are shown in Supplementary Figures S3 and S4, respectively.

To investigate 5mC methylation in *CXCR4* in human colon cancers, we extracted DNA from tumor colonocytes and adjacent normal-appearing colonocytes and tested DNA for the presence of 5mC in 3 *CXCR4* genomic loci by COBRA as illustrated in Supplementary Figure S2. Each sample was run on two lanes: lane (1) bisulfite-treated PCR amplified DNA without restriction enzyme digestion, which served as a reference control for unmethylated CpG and lane (2) bisulfite-treated PCR amplified DNA digested with a restriction enzyme recognizing amplicons containing a 5’mCpG sequence. Amplicons of region #3 showed complete digestion indicating methylation of tumor and adjacent control samples (Figure 2a).

**Figure 3:**
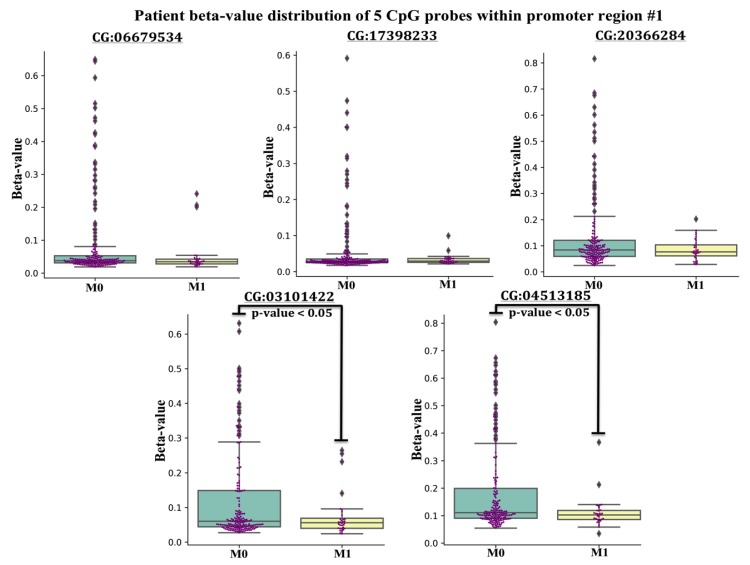
5 Boxplots displaying methylation beta-values (Y-axis) of 5 probes within promoter CpG island region #1, extracted from CRC patients classified in the Tumor, Node, Metastasis (TNM) staging system as M1 = evidence of metastasis and M0 = no evidence of metastasis from the TCGA registry. The [CG########] ID on the X-axis of each boxplot represents the unique ID of each probe within the 450K-array data set. A student *t*-test was conducted in finding mean beta-value differences for two CG probes between M0 and M1 cohorts (*p*-value < 0.05).

The lack of 5mC in regions #1 and #2 in colorectal cancers, prompted an additional study to evaluate the 5mC beta-value (methylation) distribution of 5’mCpG probes (*n* = 5) located within promoter region #1 across *n* = 371 tumors from CRC patients archived in TCGA that were classified by the Tumor, Node, Metastasis (TNM) staging system (M0 = no evidence of metastasis, M1 = evidence of metastasis). Typically, normalized 450K 5mC beta-values of CpG probes ranging from 0.00–0.19 are interpreted as unmethylated, whereas beta values ranging from 0.20–0.59 are considered partially methylated, and beta values > 0.59 are considered fully methylated [54]. The beta-distribution of CpG probes located within promoter region #1 revealed three findings. First, the vast majority of beta-values of each CpG probe were under 0.20, indicating that the promoter region #1 of *CXCR4* was unmethylated in CRC in agreement with our COBRA findings (Figure 3).

Since the vast number of CRC have low beta values in the promoter region #1, it is not surprising that we did not detect 5mC in region #1 in tumors from our patient subset. Secondly, some tumors from CRC patients had beta-values of CpG probes exceeding the 0.19 threshold, suggesting region #1 of the *CXCR4* promoter region #1 was partially methylated in a subset of CRC. Lastly, the average beta-value for 2 of 5 CpG probes was statistically lower in the metastatic (M1) cohort compared to patients without metastasis (M0), suggesting that *CXCR4* promoter hypomethylation may be occurring in metastatic CRC (Figure 3). Taken together, this suggests that hypomethylation of the *CXCR4* promoter region #1 associates with more aggressive CRC.

### 2.3. CXCR4 Promoter Hypermethylation Correlates with Decreased Expression in CRC Cell Lines

**Figure 4:**
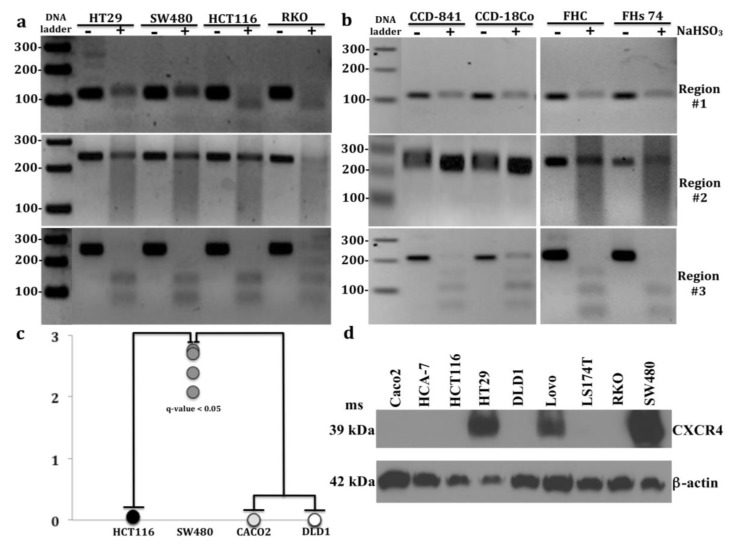
Combined bisulfite restriction analysis (COBRA) results of 3 regions of interest located in *CXCR4* from: (**a**) 4 CRC cell lines: HT29, SW480, HCT116, and RKO cells and (**b**) 4 untransformed colon cell lines: CCD-841, CCD-18Co, FHC, and FHs 74 cells. Region #3 shows complete PCR product digestion in CRC cell lines: HT29, SW480, and HCT116 cells and untransformed cell lines: FHC and FHs 74 cells, indicating that region #3 is fully methylated. Incomplete PCR product digestion in RKO cells and untransformed colon cell lines: CCD-841 and CCD-18Co cells indicate partial methylation in region #1. Incomplete PCR digestion was only evident in RKO cells in region #2 indicating partial methylation. 3 of 4 CRC cell lines: HT29, HCT116, and RKO cells displayed incomplete PCR digestion in region #1, whereas no PCR digestion was evident in any untransformed colon cell line cells. (**c**) RT-PCR analysis of *CXCR4* transcript abundance in 4 CRC cell lines: HCT116, SW480, Caco2, and DLD1 cells (*q*-value < 0.05) after multiple testing correction (Tukey post hoc test, Supplementary Table S1). Note the increase of *CXCR4* transcripts in SW480 cells compared to HCT116, Caco2, and DLD1 cells. (**d**) Western blot analysis of CXCR4 in 9 CRC cell lines: Caco2, HCA-7, HCT116, HT29, DLD1, Lovo, LS174T, RKO, and SW480 cells (Supplementary Table S2). PCR product treated with restriction enzyme lane = +; PCR product untreated lane = −.

In addition to tumors and adjacent normal colonic mucosa, we investigated CRC cell lines, HT29, SW480, HCT116, and RKO colonocytes; and untransformed colon cell lines, FHC, FHs 74, and CCD-841 colonocytes and CCD-18Co fibroblasts for the presence of 5mC in regions #1-#3. Consistent with results in primary tissues, region #3 was fully methylated in 3 of 4 CRC cell lines, HT29, SW480, and HCT116 cells and in 2 of 4 untransformed colon cell lines, FHC and FHs-74 cells (Figure 4a,b).

However, as we found only partial amplicon digestion for region #3 in CRC cell line RKO and non-transformed colon cell lines CCD-841 and CCD-18Co, we concluded that region #3 of these cell lines was only partially methylated.

Interestingly, CRC cell line RKO was the only cell line that exhibited partial methylation in region #2. Furthermore, differential methylation between CRC and untransformed colon cell lines were more pronounced in promoter region #1, where 3 of 4 CRC cell lines: HT29, HCT116, and RKO were partially methylated, whereas none of the 4 untransformed colon cell lines were methylated. Therefore, it is reasonable to suggest that methylation within promoter region #1 may be associated with CXCR4 silencing or downregulation in these 3 CRC cell lines. Increased 5mC in region #1 and little or no transcript or protein was detected in HCT116 cells and RKO cells (Figure 4c,d).

Conversely, low 5mC was detected in HT29 cells, where CXCR4 protein was expressed. Only 3 of 9 CRC cell lines had high protein expression of CXCR4: HT29, Lovo, and SW480 cells, with strongest expression in SW480 cells. Given that CXCR4 upregulation correlates with CRC metastasis and SW480 and Lovo cells possess strong migratory properties, high CXCR4 protein expression may well contribute to their migratory properties [55]. In contrast, CRC cell lines: Caco2, HCT116, and DLD1 had undetectable CXCR4 protein levels (Figure 4d) and very low *CXCR4* transcript levels (Figure 4c). Surprisingly, HT29 cells exhibited both CXCR4 protein expression and promoter methylation.

**Figure 5:**
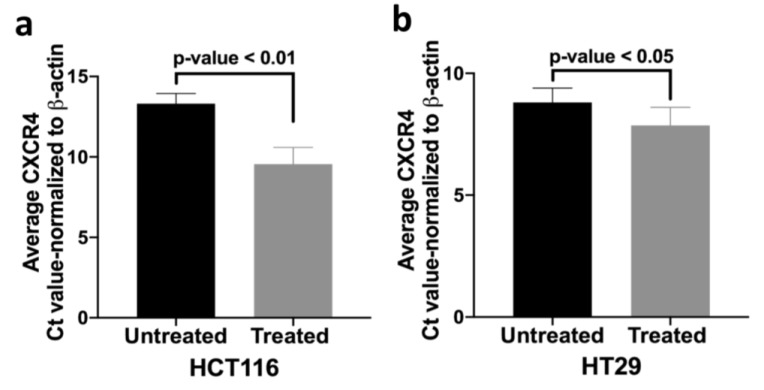
Demethylating agent 5-aza-2’deoxycytidine increases *CXCR4* transcript abundance. RT-PCR results display a decreased normalized Ct value (average) and therefore reflects the increased *CXCR4* mRNA expression in colorectal cancer cell lines (**a**) HCT116 and (**b**) HT29 after treatment with 5 µM concentration of demethylating agent 5-aza-2’-deoxycytidine.

We therefore investigated whether promoter methylation might modulate *CXCR4* expression by administering a demethylating agent (5-aza-2’-deoxycytidine) to HCT116 and HT29 cells. After 5-aza treatment there was increased *CXCR4* mRNA compared to untreated cells (Figure 5). These results suggest that promoter hypermethylation might partially inhibit *CXCR4* mRNA expression in HCT116 and HT29 cells. We speculate that increased 5mC in *CXCR4* promoter might similarly inhibit *CXCR4* expression in some tumors and control colons.

### 2.4. Increased 5hmC Deposition within CXCR4 Gene Body Correlates with Increased CXCR4 Expression

**Figure 6:**
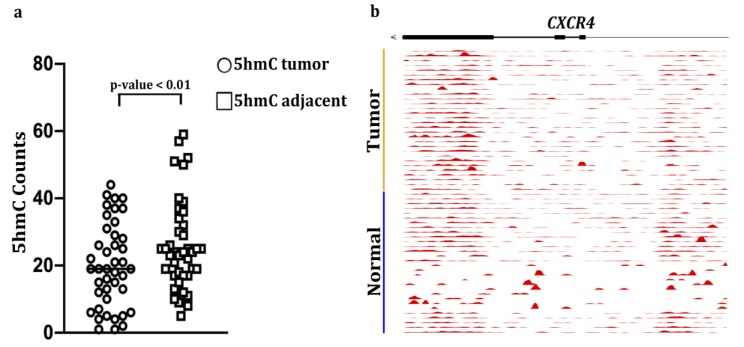
5-hydroxymethylcytosine (5hmC) abundance in *CXCR4* gene bodies in colon cancers and matched adjacent mucosa (*n* = 42 samples, *p* < 0.01, paired Student’s *t*-test): (**a**) Counts per million reads at *CXCR4* gene, (**b**) plus promoter (upstream 3kb region) in matched tumors and adjacent healthy tissue in 30 colorectal cancer patients. The moving averages at 0.01 smoother span are shown. black bars mark exons.

Since we did not detect any relationship between 5mC levels and CXCR4 expression in primary CRC vs. controls, we next examined levels of another epigenetic mark, 5hmC, in *CXCR4*. This epigenetic modification is frequently associated with active gene transcription [56]. We found that 5hmC was increased significantly in the *CXCR4* gene bodies in *n* = 42 cases of CRC compared to matched adjacent normal-appearing colonic mucosa (Figure 6a). In our CRC cohort 5hmC accumulation was most abundant within the terminal 3’ end of *CXCR4* (Figure 6b). This phenomenon has been reported in several genome-wide studies [56,57,58,59]. For example, a study by Lin et al., found that the average 5hmC read counts were consistently higher towards the 3’ end for many genes expressed in brain and liver tissues [56]. The authors speculated that a bias for 5hmC modification might begin near the end of the ORF of these genes. The underling mechanism for this bias, however, will require further study. Our observation of increased 5hmC in the *CXCR4* gene body supports the hypothesis that 5hmC modification associates with increased CXCR4 expression.

## 3. Discussion

*CXCR4* is deregulated in several cancer types and appears to be important in CRC metastasis as suggested by increased CXCR4 mRNA and protein in liver [30,37] and increased protein in lymph nodes [30] in metastatic tumors compared to primary tumors. This is further supported by increased metastatic properties in colon cancer cells following CXCR4 activation and inhibition of these characteristics with anti-CXCR4 antibodies [8]. Our studies are the first to have measured CXCR4 in matched isolated malignant colonocytes and tumor-associated stroma by Western blotting. For the first time, we also observed that *CXCR4* mRNA expression levels were higher in tumor-associated stromal cells compared to control stromal cells, suggesting that CXCR4 may play a critical role in regulating the tumor microenvironment as observed for other genes that are highly expressed in CRC-associated stromal cells [60,61]. The expression patterns of CXCR4 in our study were consistent with prior reports that analyzed CXCR4 expression in CRC. For example, RNA-seq expression profiles from a large sample of colon tumors in TCGA, revealed that *CXCR4* mRNA expression in tumors remained stable across tumor stages (I–IV). This result was consistent with two earlier studies that compared *CXCR4* mRNA expression among CRC tumor stages [30,37]. In addition, our Western blotting results showed increased CXCR4 expression in tumor colonocytes compared to matched adjacent normal-appearing colonocytes, and were in agreement with Ottaiano and colleagues’ findings of increased CXCR4 in malignant colonocytes as assessed by immunostaining [8].

Of the cell lines examined in the report, N = 6 of 9 CRC cell lines: RKO, HCT116, LS174T, Lovo, DLD1, and HCA-7 cells exhibit a hypermutable phenotype and are classified as micro-satellite instable (MSI) tumor cell lines, whereas the remaining three CRC cell lines: HT29, SW480, and Caco2 cells are classified as microsatellite stable (MSS) tumor cell lines. The microsatellite unstable phenotype is associated with poor overall survival and is caused by biallelic loss of a DNA mismatch repair enzyme, which accounts for about 15% of all sporadic CRCs [62,63]. One study concluded that overall most genes were downregulated in MSI tumors, but noted upregulation of mucin-related genes as a key molecular biomarker characterizing MSI tumors [64]. Interestingly, CXCR4 protein expression in microsatellite unstable CRC cell lines was detected only in Lovo cells, which represents 1 of 6 (17%) MSI tumors assayed; but CXCR4 was detected in 2 of 3 (67%) of MSS tumors assayed: HT29, SW480 cells. Further studies comparing primary MSI and MSS tumors will be needed to determine whether CXCR4 expression levels differ between these tumor types.

To examine potential epigenetic mechanisms regulating *CXCR4* in CRC, we assessed 5mC modifications in *CXCR4* by COBRA analysis, TCGA methylation data, and 5hmC modifications by publicly available nano-hmC-Seal data. The 5mC portion of the study was initially prompted by the fact that the 450 K methylation data from the UCSC Genome Browser identified *CXCR4* promoter hypermethylation in HCT116 and Caco-2 cells, which lacked CXCR4 protein expression. In addition, the 5mC pattern spanning *CXCR4* in HCT116 and Caco-2 cells served as a template in identifying potential regulatory hotspots to test for the presence/absence of 5mC in CRCs. In turn, regions #1 and #2 were identified as both regions overlap a CpG island within the promoter or a 5’ regulatory region in *CXCR4* and are therefore most likely to impact the transcription status of *CXCR4*. The COBRA technique was chosen in that it allowed for a greater number CpGs to be evaluated in regions #1 and #2 (*n* = 9) compared to other forms of methylation-interrogating end-point PCR techniques like methylation-specific PCR (MSP).

In our sample set of CRC tissues we did not observe decreased 5mC in *CXCR4* despite increased mRNA and protein expression in these tumors. Upon analysis of CRC tumor methylation in the TCGA, we found that promoter *CXCR4* 5mC hypermethylation exists in a small subset of primary colon cancers and those primary tumors with metastasis exhibit loss of 5mC in the promoter region. Recent studies of TCGA for both DNA 5’mCpG methylation and RNA-seq analysis suggest that 5’mCpG methylation could exert gene-specific positive or negative effects on gene transcription [65,66]. We also found increases in 5hmC in the *CXCR4* gene body in an independent cohort of colon tumors [52], consistent with 5hmC serving as a marker of active gene transcription. A limitation of our 5hmC analysis is that expression levels of CXCR4 mRNA and protein were not available for this cohort and the TCGA cohort did not have 5hmC analysis.

Although, the mechanisms underlying aberrant methylation patterns in cancer remain incompletely understood, global hypomethylation, associated with genomic instability and promoter hypermethylation of tumor suppressor genes are observed in many tumors, including colon cancer [41,42,43,44,45,46]. The latter may be related to increased enzymatic activity of DNA methyltransferases in CRC compared to normal colonic mucosa [67]. Whether changes in 5hmC and/or 5mC promoter methylation regulate CXCR4 expression in metastatic CRC will require additional studies.

In CRC cell lines our results suggest that 5mC present within CpG island region #1 of the promoter may contribute to *CXCR4* gene silencing in HCT116 and RKO cells as negligible levels of CXCR4 mRNA and no protein were detected in HCT116 cells and no CXCR4 protein was detected in RKO cells. Indeed, our COBRA findings of 5mC located in the CpG rich region #1 of the *CXCR4* promoter in HCT116 cells are consistent with *CXCR4* promoter methylation data in HCT116 cells available in the UCSC Genome Browser. While CXCR4 was still expressed in HT29 cells, despite *CXCR4* promoter methylation, our demethylation studies suggest that DNA methylation limits CXCR4 expression in these cells. In this regard, treating both HT29 and HCT116 cells with DNA hypomethylating agent (5-aza) increased *CXCR4* mRNA. A previous study in MCF7 breast cancer cells also demonstrated that 5-aza treatment demethylated the *CXCR4* promoter and concomitantly increased *CXCR4* mRNA expression [68]. We speculate that similar demethylation-induced changes in the *CXCR4* promoter are occurring in (5-aza) treated HT29 and HCT116 cells that showed increased *CXCR4* mRNA. Persistent CXCR4 gene expression in HT29 cells might result from lower levels of 5mC methylation in region #1 or in other *CXCR4* promoter regions compared to those of HCT116 and RKO cells. We were only able to interrogate one CpG site in region #1 and in the promoter region, because of design limitations of suitable primers, thereby limiting the number of promoter associated CpG islands tested in this study. Future studies will also address potential differences in 5hmC in the *CXCR4* promoters in these cell lines that could also contribute to differences in CXCR4 expression

## 4. Materials and Methods

### 4.1. Human Tissue Collection

Human colonic adenocarcinomas and adjacent normal appearing colonic mucosa were obtained for the Department of Surgical Pathology at The University of Chicago under an approved IRB protocol (10-209-A). Resected tissues were placed in an ice bath and transported promptly to the Surgical Pathology Department. Representative tumor sections and adjacent normal appearing colonic mucosa were dissected free of underlying muscle. Care was taken to avoid areas with visible necrosis. Colonocytes and stromal cells were isolated as previously described and purities assessed by cytokeratin20 (colonocyte marker) and vimentin (stromal cell marker) Western blotting [69]. Normal colon tissues were obtained from colon biopsies taken 20 cm from the anus under the approved IRB 10-209-A. For DNA methylation studies, biopsies were stored in RNA later that also preserves DNA.

### 4.2. Isolation of Coloncytes and Stromal Cells from Colonic Mucosa

Colonocytes and stromal cells were isolated following previously published methods with minor modifications [70,71,72]. Briefly, colon tissues were removed and mucosa scrape-isolated and minced with razor blade into 2 mm fragments that were collected in tubes containing 6 mL sterile ice-cold transport media, 50 IU/mL penicillin (Millipore Sigma, St. Louis, MO, USA), 50 µg/mL streptomycin (Millipore Sigma, St. Louis, MO, USA) and 0.5 mg/mL gentamycin (Millipore Sigma, St. Louis, MO, USA). Tissue was washed 3 times by gentle inversion and collected by gravity sedimentation and re-suspended in 10 mL chelating buffer (transport media plus 1 mM EDTA (Millipore Sigma, St. Louis, MO, USA) and 1 mM EGTA (Millipore Sigma, St. Louis, MO, USA). Tissue was incubated on a shaker overnight at 4 °C to release colonocytes into the supernatant. The pellet was washed three times with 3 mL ice-cold PBS (Thermo Fisher Scientific, Waltham, MA, USA), releasing residual colonocytes, and colonocyte-containing supernatants were combined. Supernatants containing the epithelial cell fraction, and pellets containing the stromal cell fraction were centrifuged at 400× *g* for 5 min at 4 °C, yielding loosely packed pellets from which proteins were solubilized in 2× Laemmli buffer (Millipore Sigma, St. Louis, MO, USA).

### 4.3. Cell Culture

All colon cancer and normal colon cell lines were obtained from ATCC and cultured at 37 °C in a humified atmosphere of 95% O_2_ and 5% CO_2_ and 10% fetal calf serum (Thermo Fisher Scientific, Waltham, MA, USA). Cell authentications for all lines were confirmed by short tandem repeats by (IDEXX Bioanalytics, Columbia, MO, USA). Cells were shown to be mycoplasma free by IDEXX. HCT116 and FHC cells were grown in high glucose DMEM medium (Thermo Fisher Scientific, Waltham, MA, USA). DLD-1 cells were cultured in RPMI-1640 media (ATCC, Manassas, VA, USA). SW480 cells were grown in ATCC-formulated Leibovitz’s L-15 Medium (ATCC, Manassas, VA, USA). RKO, LS-174T, Caco2, CCD-841, CCD-18Co cells were grown in ATCC-formulated Eagle’s Minimum Essential Medium (ATCC, Manassas, VA, USA). Lovo cells were cultures in ATCC-formulated F-12K Medium (ATCC, Manassas, VA, USA). FHs 74 cells were grown in ATCC-formulated Hybri-Care medium (ATCC, Manassas, VA, USA). HCA-7 cells, derived from a rectal carcinoma {Marsh, 1993 #1850}, were generously provided by Dr. Susan Kirkland (ICRF, London UK). Cells were grown in McCoy’s media (ATCC, Manassas, VA, USA).

### 4.4. Measurement of CXCR4 Steady-State Transcript Levels

mRNA was extracted from colonocytes and stromal cells isolated from tumors and control colon (CRC *n* = 6; control colons *n* = 4) and from CRC cell lines: HCT116, SW480, Caco2, DLD1 and cells using the RNeasy^®^ Mini Kit (Qiagen, Germantown, MD, USA). mRNA (150 ng) was then reverse transcribed into cDNA using High Capacity cDNA Reverse Transcription Kit (Applied Biosystems, Beverly, MD, USA) in 20 μL total volume. Incubation conditions were 37 °C for 60 min, and 95 °C for 5 min. The resulting first-strand cDNA was used as template for quantitative PCR in triplicate using Fast SYBR Green QPCR Master Mix kit (Applied Biosystems, Beverly, MD, USA). Reverse transcribed cDNA (1:10 dilution) and primers were mixed in 20 µL with fast SYBR green master mixture. Reactions were monitored on the LightCycler^®^ 480 II System (Roche Diagnostic Corporation, Basel, Switzerland). Reactants were initially heated to 95 °C for 20 s followed by 40 cycles: denaturation step at 95 °C for 10 s, annealing step at 55 °C for 15 s and extension step at 60 °C for 30 s. PCR amplification was verified by melting curve and electrophoretic analyses of the PCR products on a 2% agarose gel. Negative control reactions were also included (omitting reverse transcriptase or template cDNA). Data were analyzed using the comparative 2^(-ΔΔCt) method [73] and mRNA levels were normalized to β-actin and expressed as fold-control (Supplementary Table S1). The multiple comparisons analysis using Tukey’s method (*q* < 0.05) consisted of 11 groups, including: human whole tissue, control colonocytes and stromal cells, colonocytes and stromal cells from normal appearing mucosa adjacent to tumor, tumor colonocytes and tumor stromal cells. *CXCR4* expression was also assessed in 4 CRC: HCT116, SW480, Caco2, and DLD1 cell lines. Primer sequences for quantitative RT-PCR include:

*β-actin* Forward primer (5’->3’) CAGCCATGTCGTTGCTATCCAGG;

*β-actin* Reverse primer (5’->3’) AGGTCCAGACGCAGGATGGCATG;

*CXCR4* Forward primer (5’->3’) GCGGTTACCATGGAGGGGAT;

*CXCR4* Reverse primer (5’->3’) CCCATGACCAGGATGACCAAT;

### 4.5. CXCR4 Western Blotting

Western blotting was performed to assess CXCR4 expression in primary colon cancers and adjacent normal appearing colonic mucosa, and 9 colorectal cell lines: Caco2, HCT116, HCA-7, HT29, DLD1, Lovo, LS174T, RKO, and SW480 colonocytes. Proteins were extracted in SDS-containing Laemmli buffer, quantified by RC-DC protein assay and subjected to Western blotting. Sample lysates (60 μg protein) and pre-stained molecular weight markers were separated by SDS-PAGE on 4%–20% resolving polyacrylamide gradient gels and electroblotted to PVDF membranes. Blots were incubated overnight at 4 °C with specific primary rabbit monoclonal antibody at 1:1000 dilution (CXCR4, Abcam ab181020, Cambridge, MA, USA) followed by 1hr incubation with appropriate peroxidase-coupled secondary antibodies. CXCR4 Abcam Cat#ab181020 Blocking: 5% milk for 3 h 1°AB 1:1000 in 5% milk overnight at 4 °C secondary AB 1:3000 anti-rabbit antibodies in 5% milk for 1 h at RT that were detected by enhanced chemiluminescence using X-OMAT film (Carestream, Rochester, NY, USA). Xerograms were digitized using Epson Perfection V600I scanner (Epson, Long Beach, CA, USA). and CXCR4 band intensity was quantified using Un-Scan-It software V6.3 (Silk Scientific Inc., Orem, UT, USA) [69] (Supplementary Table S2). A paired *t*-test was conducted to determine CXCR4 band intensity differences between matched CRC tumor and adjacent tissue samples.

### 4.6. Combined Bisulfite Restriction Analysis

Prior to COBRA [53], genomic DNA (gDNA) was extracted and eluted in water from human tissue samples and colorectal cell lines (HCT116, RKO, HT29, and SW480) using the DNeasy Blood and Tissue Kit (Qiagen, Germantown, MD, USA). Clinical information for patients for the methylation studies is summarized in Supplementary Table S3. Briefly, gDNA (~200 ng) was treated with bisulfite using the EZ DNA Methylation-Gold Kit (Zymo, Irvine, CA, USA) per the manufacturer’s protocol. Bisulfite-treated DNA was eluted in water. Bisulfite converted DNA (~200 ng) of bisulfite converted DNA was used as template under standard thermal conditions (15 min hot start (94 °C denaturation for 30 s, 56 °C annealing temp, and 72 °C extension for 40 cycles) 72 °C final extension for 10 min) in a 25 μL reaction using the PyroMark PCR kit (Qiagen, Germantown, MD, USA) according to the manufacturer’s instructions. The bisulfite-specific primers with sequences designed by MethPrimer software [74] include:

Region #1: Promoter CpG island upstream of *CXCR4* TSS [1 CpG] HpyCH4IV

Forward primer: (5’>3’)TTTTGAGTAGAGGATAAGTTTTGGTA;

Reverse primer: (5’->3’) ACAAACCTCAACCAATCTAAAATC;

Region #2: Intragenic CpG island at the 5’ regulatory end of *CXCR4* [8 CpGs] BstUI

Forward primer: (5’>3’)GGTGGTTATTGGAGTATTTAGGTTTT;

Reverse primer: (5’->3’)AACAAAATCCCTAAACTTC;

Region #3: Terminal 3’ exon of *CXCR4* [2CpGs] HpyCH4IV

Forward primer: (5’->3’)TTATTTATAAGTTATTGGGGTAGAAG;

Reverse primer: (5’->3’)ATCCTAACCTTCATCAATCTAAAC;

PCR products (~7 μL) were digested in a 25 μL reaction containing either BstUI (60 °C) or HpyCH4IV (37 °C) restriction enzyme for 4 h. Restriction digests were then resolved on a 2.5% agarose gel with ethidium bromide. BstUI: cuts 5’CGCG’3 sequence and HpyCH4IV: cuts 5’ACGT’3 sequence.

### 4.7. 5hmC-Modified Locations in the CXCR4 Gene Body

5hmC-Seal data were downloaded for 42 pairs of tumor (TU) and adjacent tissue (TI) samples from patients with colorectal cancer (GSE89570) [52]. Raw sequencing reads were summarized for the gene bodies or promoters according to the current GENCODE annotations (release 19). The normalized counts from DESeq2 by library size were log2 transformed and corrected for batch effect using linear regression. The paired t test was used to evaluate whether the 5hmC modification levels in *CXCR4* gene body were different between tumors and adjacent tissues (*p*-value < 0.01).

### 4.8. Inhibition of 5mC Deposition in Colorectal Cancer Cell Lines HCT116 and HT29

HCT116 and HT29 cells were seeded at ~10,000 cells at day -2. On day 0, culture medium was removed and replaced with fresh medium containing 5µM 5’-aza-2’-deoxycytidine (Millipore Sigma, St. Louis, MO, USA) dissolved in DMSO and incubated for 72 h. On day 3, cells were rinsed twice with PBS and harvested for RNA and proteins. *CXCR4* mRNA expression was analyzed by RT-PCR as previously described.

### 4.9. CXCR4 Bioinformatics Analysis

High-throughput data from tumors from CRC patients were extracted from The Cancer Genome Atlas (TCGA) and utilized for dissecting relationships among 5mC methylation, mRNA gene expression and clinical correlates [75]. CRC were categorized into respective TNM (M0, M1) or tumor stages (I–IV) for analysis. A python statistical data visualization library (Seaborn) was used to generate boxplots showing mRNA expression and methylation distributions [76]. The x-axis represents the TNM staging classification of CRC and the y-axis represents FPKM (mRNA gene expression) values from RNA-seq data or beta (methylation) values from 450K methylation microarray probes spanning two CpG islands within *CXCR4* (Supplementary Table S4).

## 5. Conclusions

In summary, we confirmed upregulation of CXCR4 in CRC and extended prior studies to show by cell-specific isolation and Western blotting that malignant colonocytes in CRC have the highest expression levels. Furthermore, for the first time, we demonstrate that tumor-associated stromal cells express increased *CXCR4* mRNA. These changes in CXCR4 expression were not accompanied by changes in 5mC, but rather by previously unreported increased 5hmC that we postulate marks this gene for increased transcription in CRC. Multiple mechanisms regulating CXCR4 expression in CRC are likely involved, including DNA epigenetic processes such as increases in 5hmC in the *CXCR4* gene body [52], acquisition of activating and loss of repressive histone marks such as increased H3K4me3 and decreased H3K27me3, respectively, [77]; and recruitment of trans-activating factors such as nuclear respiratory factor1 (NRF1) [78]. Additional studies to discover new or novel genetic and epigenetic mechanisms regulating *CXCR4* are warranted to potentially target CXCR4 in colon cancer.

## Supplementary Materials

The following are available online at https://www.mdpi.com/2072-6694/12/3/539/s1, Figure S1: Normalized CXCR4 FPKM values from CRC patients categorized by tumor stage, Figure S2: UCSC Genome Browser (hg19 build) depiction of CXCR4 containing 2 CpG islands (green) and 3 Methyl 450K probe sub tracks for colorectal cell lines Caco2 and HCT116 (Probe colors of Blue = unmethylated, Purple = partially methylated, and Yellow = fully methylated), Figure S3: Full-length COBRA images from Figure 2a (T tumor, C control), Figure S4: Full-length COBRA images from healthy control patients from Figure 2b (H# human subject number; P proximal colon, D distal colon), Table S1: Clinical characteristics and statistical analysis of patients used in the qPCR studies, Table S2: Densitometry statistics of CXCR4 bands in Figure 1c and Figure 4d. Table S3: Clinical characteristics of patients used in the COBRA analysis in Figure 2 and Figures S3 and S4. Table S4: Number of patients deposited in TCGA that were used in the CXCR4 RNA-seq and 450K methylation microarray analysis.
