# Correction: Stuckel, et al.; Enhanced CXCR4 Expression Associates with Increased Gene Body 5-Hydroxymethylcytosine Modification but Not Decreased Promoter Methylation in Colorectal Cancer. *Cancers* 2020, *12*, 539

**DOI:** 10.3390/cancers12113104

**Published:** 2020-10-23

**Authors:** Alexei J. Stuckel, Wei Zhang, Xu Zhang, Shuai Zeng, Urszula Dougherty, Reba Mustafi, Qiong Zhang, Elsa Perreand, Tripti Khare, Trupti Joshi, Diana C. West-Szymanski, Marc Bissonnette, Sharad Khare

**Affiliations:** 1 Division of Gastroenterology and Hepatology, Department of Medicine, University of Missouri, Columbia, MO 65212, USA; ajsn6c@mail.missouri.edu (A.J.S.); beefjiao@gmail.com (Q.Z.); elpxc9@mail.missouri.edu (E.P.); kharet@health.missouri.edu (T.K.); 2 Department of Preventive Medicine and The Robert H. Lurie Comprehensive Cancer Center, Northwestern University Feinberg School of Medicine, Chicago, IL 60611, USA; wei.zhang1@northwestern.edu; 3 Department of Medicine, University of Illinois, Chicago, IL 60607, USA; zhangxu@uic.edu; 4 Bond Life Sciences Center, University of Missouri, Columbia, MO 65201, USA; zengs@mail.missouri.edu (S.Z.); joshitr@health.missouri.edu (T.J.); 5 Department of Electrical Engineering and Computer Science, University of Missouri, Columbia, MO 65201, USA; 6 Section of Gastroenterology, Hepatology and Nutrition, Department of Medicine, The University of Chicago, Chicago, IL 60637, USA; udougher@medicine.bsd.uchicago.edu (U.D.); rmustafi@uchicago.edu (R.M.); dcwest@bsd.uchicago.edu (D.C.W.-S.); mbissonn@medicine.bsd.uchicago.edu (M.B.); 7 Institute for Data Science and Informatics, University of Missouri, Columbia, MO 65211, USA; 8 Department of Health Management and Informatics, School of Medicine, University of Missouri, Columbia, MO 65212, USA; 9 Harry S. Truman Memorial Veterans’ Hospital, Columbia, MO 65201, USA

The authors would like to make a correction to their published paper [1], Figure 6a.

On page 9 the legend terms of Figure 6a denoted by a circle “5hmC tumor” and square “5hmC adjacent” were switched by accident and were switched back to where “5hmC tumor” is accompanied by a square and “5hmC adjacent” accompanied by a circle.

The original version of Figure 6a is:

**Figure 6:**
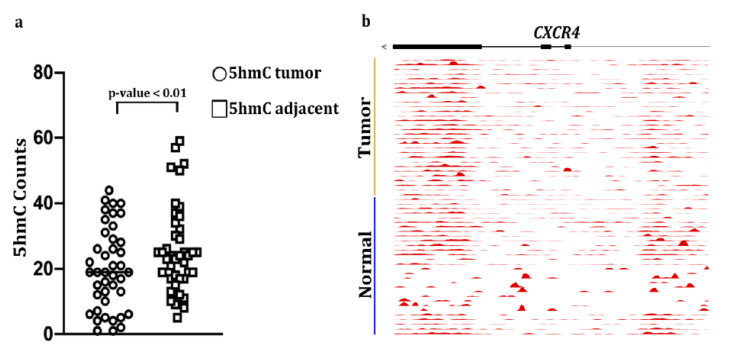
5-hydroxymethylcytosine (5hmC) abundance in CXCR4 gene bodies in colon cancers and matched adjacent mucosa (n = 42 samples, *p* < 0.01, paired Student’s *t*-test): (**a**) Counts per million reads at CXCR4 gene, (**b**) plus promoter (upstream 3kb region) in matched tumors and adjacent healthy tissue in 30 colorectal cancer patients. The moving averages at 0.01 smoother span are shown. Black bars mark exons.

and should be replaced with the following Figure 6a:

**Figure 6:**
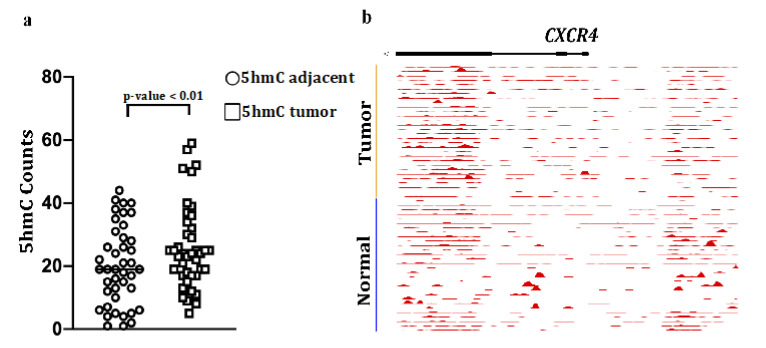
5-hydroxymethylcytosine (5hmC) abundance in CXCR4 gene bodies in colon cancers and matched adjacent mucosa (n = 42 samples, *p* < 0.01, paired Student’s *t*-test): (**a**) Counts per million reads at CXCR4 gene, (**b**) plus promoter (upstream 3kb region) in matched tumors and adjacent healthy tissue in 30 colorectal cancer patients. The moving averages at 0.01 smoother span are shown. Black bars mark exons.

We stress that this correction does not change the written portion of the figure legend, interpretation of results, or final conclusion of this manuscript. The manuscript will be updated and the original will remain available on the article webpage. The authors would like to apologize for any inconvenience caused.
