# Supplementary material for: Enhanced CXCR4 Expression Associates with Increased Gene Body 5-Hydroxymethylcytosine Modification but not Decreased Promoter Methylation in Colorectal Cancer
Source: Cancers (Basel). 2020 Feb 26;12(3):539. doi: 10.3390/cancers12030539 (PMC7139960; doi:10.3390/cancers12030539)
Supplement: Supplementary file 1 [file cancers-12-00539-s001.zip › Figure S3.docx]

**C#492**

**T#492**

**T#499**

**T#520**

**C#520**

**T#549**

**C#549**

**T#534**

**C#534**

**T#535**

**C#535**

**-**

**-**

**-**

**+**

**+**

**+**

**-**

**-**

**-**

**-**

**+**

**+**

**+**

**+**

**-**

**-**

**-**

**-**

**+**

**+**

**+**

**+**

**-**

**-**

**+**

**+**

**+**

**+**

**+**

**+**

**+**

**+**

**-**

**-**

**-**

**-**

**-**

**-**

**T#520**

**C#520**

**T#549**

**C#549**

**100bp ladder**

**300-**

**-**

**-**

**+**

**+**

**+**

**+**

**+**

**+**

**+**

**+**

**-**

**-**

**-**

**-**

**-**

**-**

**300-**

**200-**

**100-**

**100bp ladder**

**100bp ladder**

**100bp ladder**

**100bp ladder**

**100bp ladder**

**100bp ladder**

**100bp ladder**

**100bp ladder**

**100bp ladder**

**100bp ladder**

**100bp ladder**

**T#511**

**C#511**

**T#517**

**C#517**

**T#518**

**C#518**

**T#519**

**C#519**

**T#520**

**C#520**

**T#549**

**C#549**

**C#535**

**T#534**

**T#535**

**-**

**-**

**-**

**+**

**+**

**+**

**-300**

**-200**

**-100**

**-**

**-**

**-**

**+**

**+**

**+**

**Region #3**

**Region #2**

**Region #1**

**-100**

**-100**

**-100**

**-100**

**-100**

**100-**

**100-**

**100-**

**100-**

**100-**

**-200**

**-200**

**-200**

**-200**

**-200**

**200-**

**200-**

**200-**

**200-**

**200-**

**C#492**

**T#492**

**T#499**

**-300**

**-300**

**300-**

**300-**

**-300**

**-300**

**-300**

**300-**

**300-**

**T#511**

**C#511**

**T#517**

**C#517**

**T#518**

**C#518**

**T#519**

**C#519**

**-**

**-**

**-**

**+**

**+**

**+**

**-**

**-**

**-**

**-**

**+**

**+**

**+**

**+**

**-**

**-**

**-**

**-**

**+**

**+**

**+**

**+**

**T#534**

**C#534**

**T#535**

**C#535**

**-**

**-**

**-**

**-**

**+**

**+**

**+**

**+**

**-**

**-**

**+**

**+**

**+**

**+**

**+**

**+**

**+**

**+**

**-**

**-**

**-**

**-**

**-**

**-**

**C#492**

**T#492**

**T#499**

**T#511**

**C#511**

**T#517**

**C#517**

**T#518**

**C#518**

**T#519**

**C#519**
