# Supplementary figures and images for: Enhanced CXCR4 Expression Associates with Increased Gene Body 5-Hydroxymethylcytosine Modification but not Decreased Promoter Methylation in Colorectal Cancer
Source: Cancers (Basel). 2020 Feb 26;12(3):539. doi: 10.3390/cancers12030539 (PMC7139960; doi:10.3390/cancers12030539)

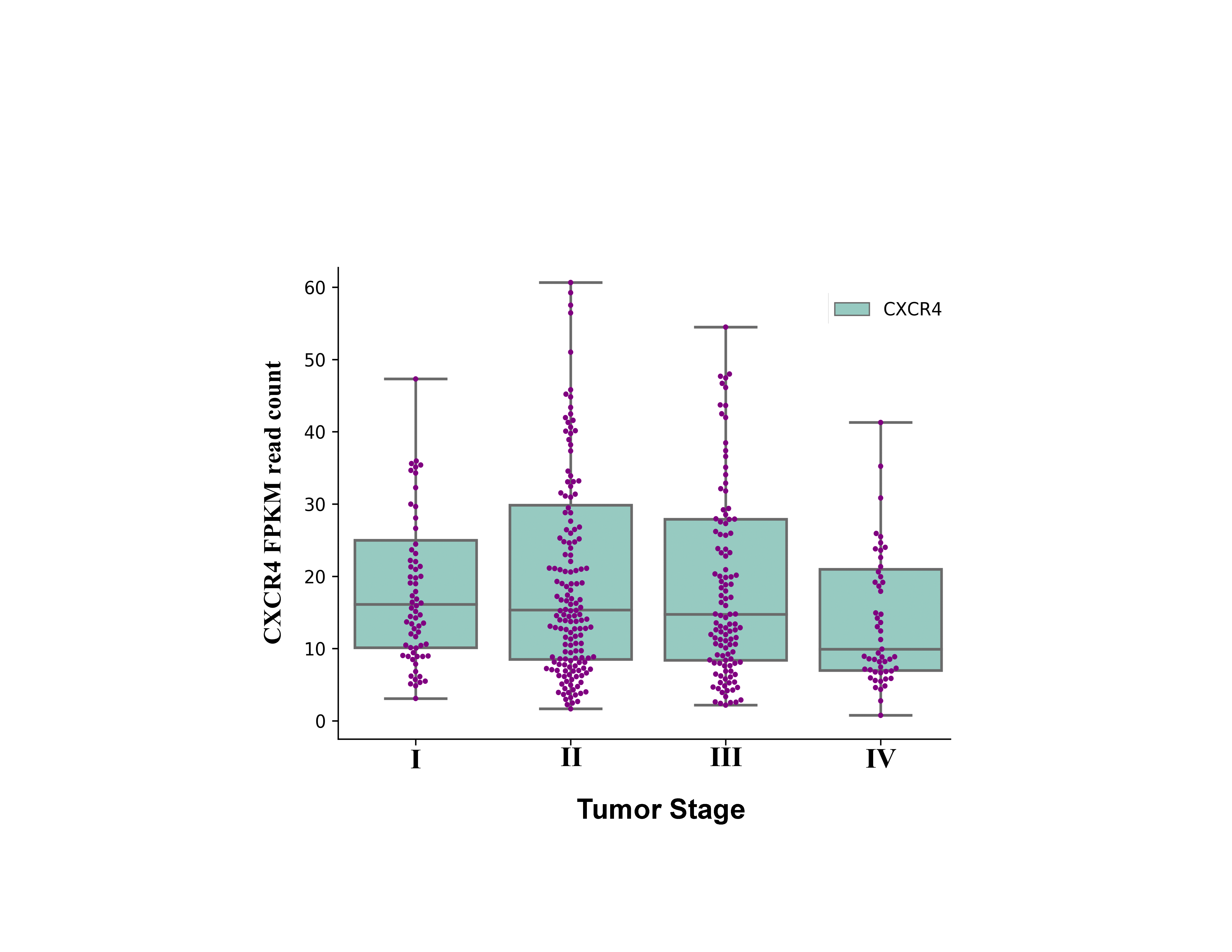

Supplement: Supplementary file 1 [file cancers-12-00539-s001.zip › Figure S1.docx]

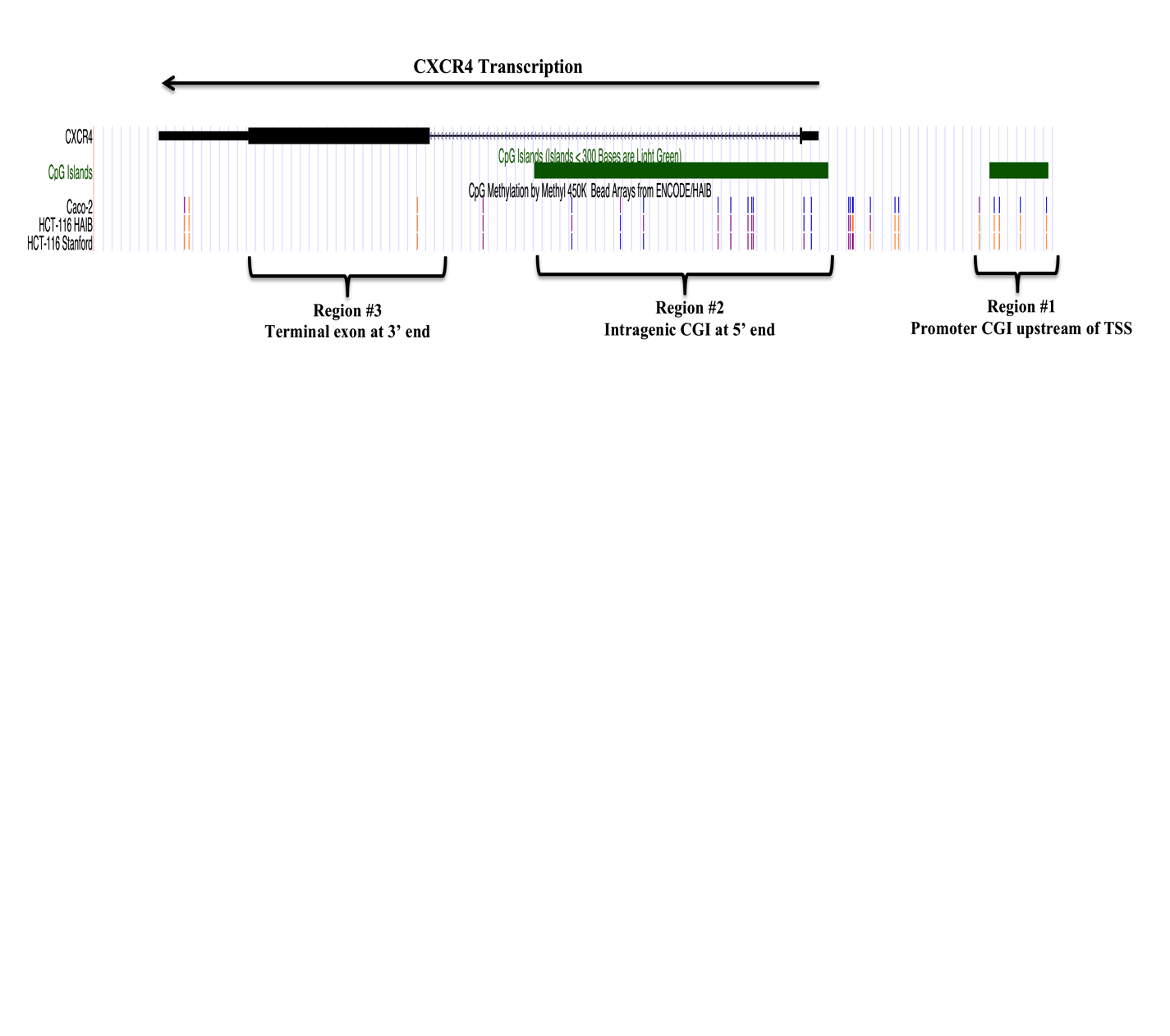

Supplement: Supplementary file 1 [file cancers-12-00539-s001.zip › Figure S2.docx]

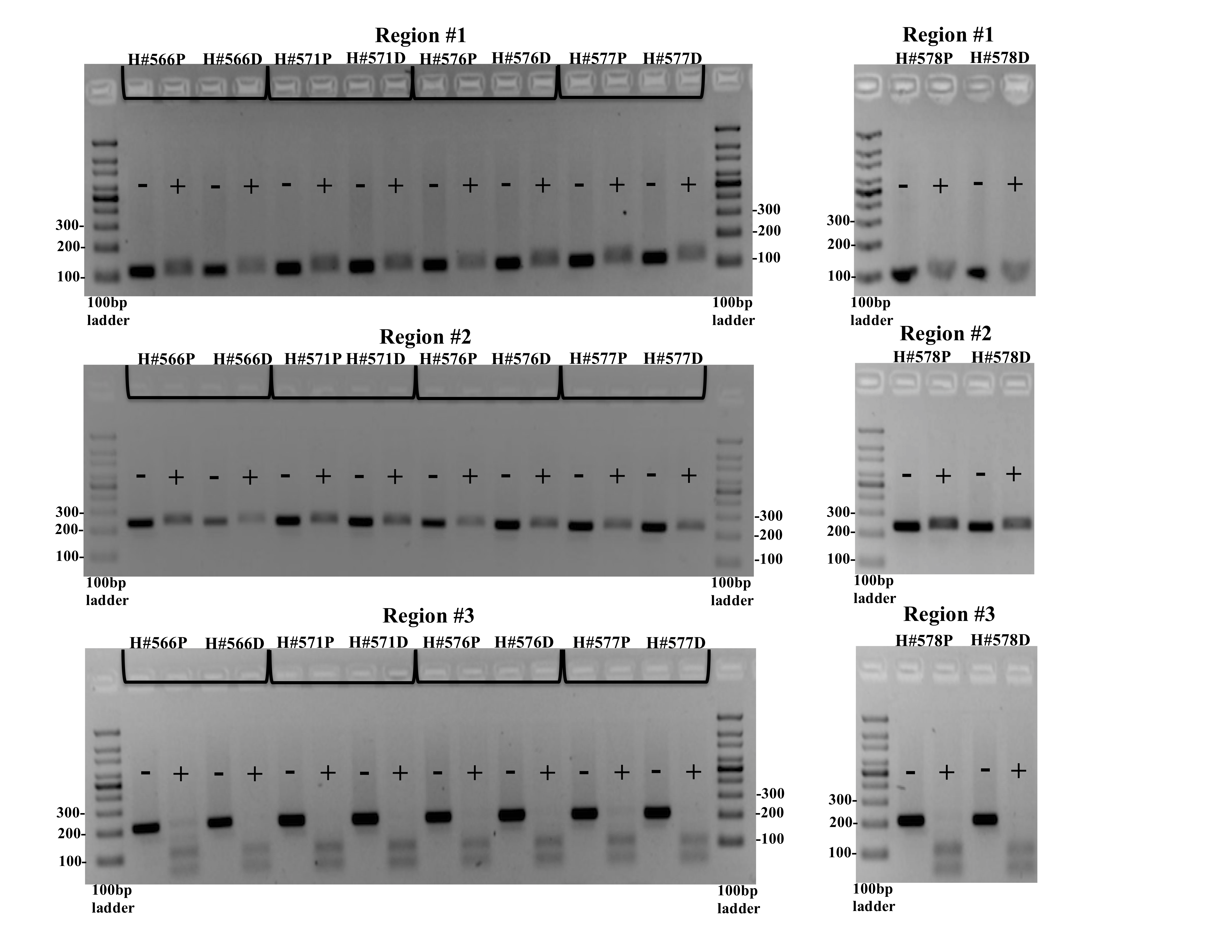

Supplement: Supplementary file 1 [file cancers-12-00539-s001.zip › Figure S4.docx]

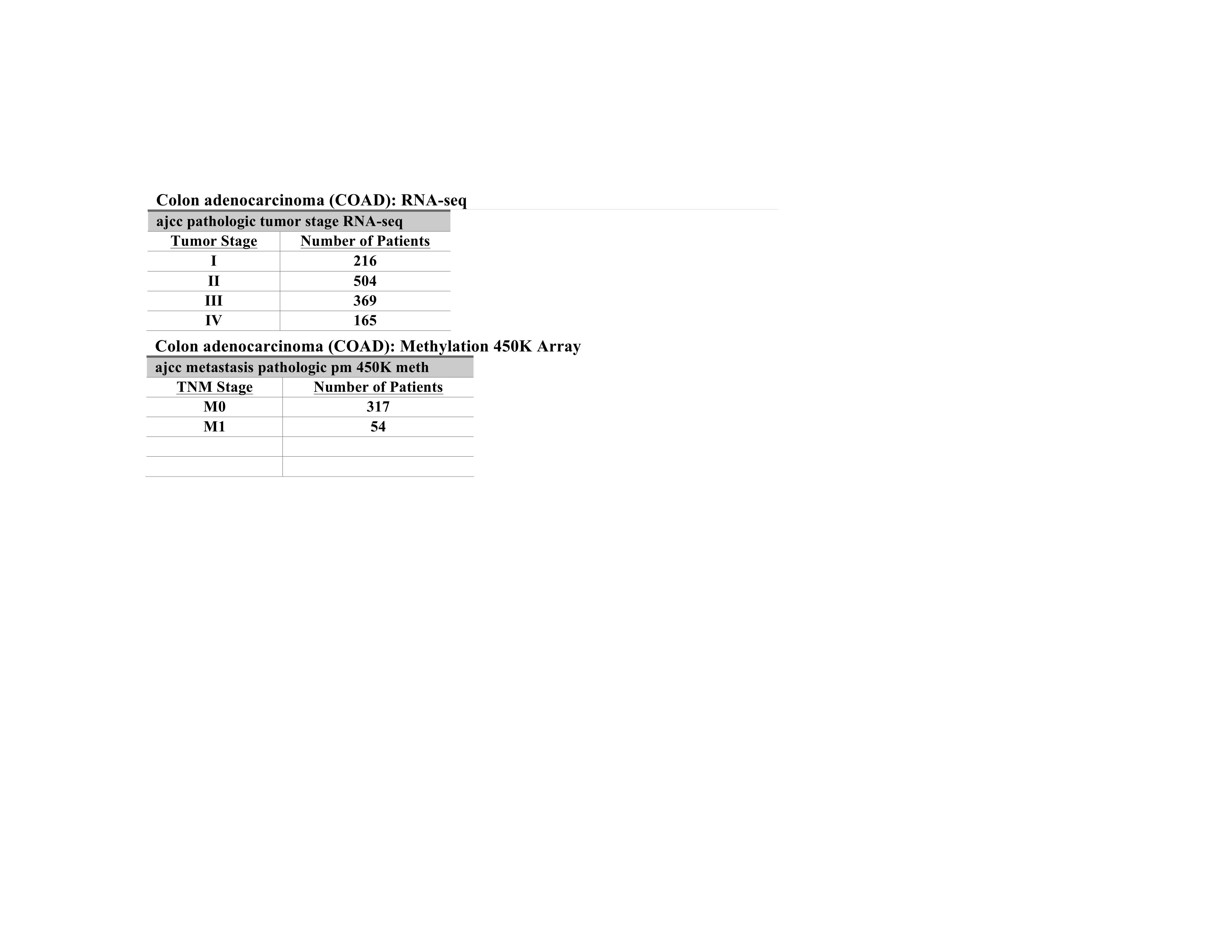

Supplement: Supplementary file 1 [file cancers-12-00539-s001.zip › Table S4.docx]
